# Dietary Principles, Interventions and Oxidative Stress in Psoriasis Management: Current and Future Perspectives

**DOI:** 10.3390/medicina61071296

**Published:** 2025-07-18

**Authors:** Oana-Georgiana Vaduva, Aristodemos-Theodoros Periferakis, Roxana Elena Doncu, Vlad Mihai Voiculescu, Calin Giurcaneanu

**Affiliations:** 1 Faculty of Medicine, “Carol Davila” University of Medicine and Pharmacy, 050474 Bucharest, Romania; 2 Elkyda, Research & Education Centre of Charismatheia, 17675 Athens, Greece; 3 Dermatology Department, Elias Emergency University Hospital, 011461 Bucharest, Romania

**Keywords:** psoriasis, nutrition, diet, oxidative stress, vitamin D, supplements

## Abstract

Psoriasis is a chronic inflammatory autoimmune disease that causes significant deterioration of the quality of life, and due to its multifactorial causes, it is often difficult to manage. Apart from genetic and environmental components, an important part of its pathophysiology comprises an oxidative stress induction that the standard antioxidative mechanisms of the human body cannot compensate for. Moreover, in many psoriatic patients, there is a documented imbalance between antioxidant and pro-oxidative factors. Usually, psoriasis is evaluated using the Psoriasis Area and Severity Index (PASI) score. It has been demonstrated that dietary choices can lead to significant modification of PASI scores. Hypocaloric diets that are rich in antioxidants are highly effective in this regard, especially when focusing on vegetables and restricting consumption of animal-derived protein. Specific dietary regimens, namely the Mediterranean diet and potentially the ketogenic diet, are very beneficial, in the former case owing in large part to the omega-three fatty acids it provides and its ability to alter gut microbiome, a factor which seems to play a notable role in the pathogenesis of the disease. Another option is the topical application of vitamin D and its analogues, combined with corticosteroids, which can ameliorate the manifestations of psoriasis at the level of the skin. Finally, oral vitamin D supplementation has a positive impact on psoriatic arthritis and can mitigate the risk of associated comorbidities.

## 1. Introduction

In recent years there has been an increased incidence of autoimmune pathologies, and this has led to an heightened awareness of the associated morbidity as well as psychological and mental challenges. One of the most well-known autoimmune diseases is psoriasis, a complex chronic immune-mediated disease presenting with a variety of manifestations, of which the most prominent are usually the cutaneous ones, although other locations and systems may be affected as well [1,2,3,4].

Currently, it is estimated that psoriasis affects over 120 million people at a worldwide level, with incidence and prevalence showing great geographical variations [5]. Notably, it seems that higher-income countries and people of Caucasian ancestry are at higher risk of developing psoriasis [6]. The average prevalence is around 8% in the adult population [6], while, based on recent research results, the actual burden of disease is higher due to underdiagnosis of psoriasis-related morbidities like psoriatic arthritis [7]. The systemic inflammatory nature of the disease means that patients usually exhibit numerous comorbidities [8,9], which in turn decrease their quality of life [10,11,12]. The most common signs of the disease are red and scaly plaques, which usually cause some degree of itching and discomfort [13]; other comorbidities, of varying degrees of severity, are also associated with psoriasis [14].

**Figure 1:**
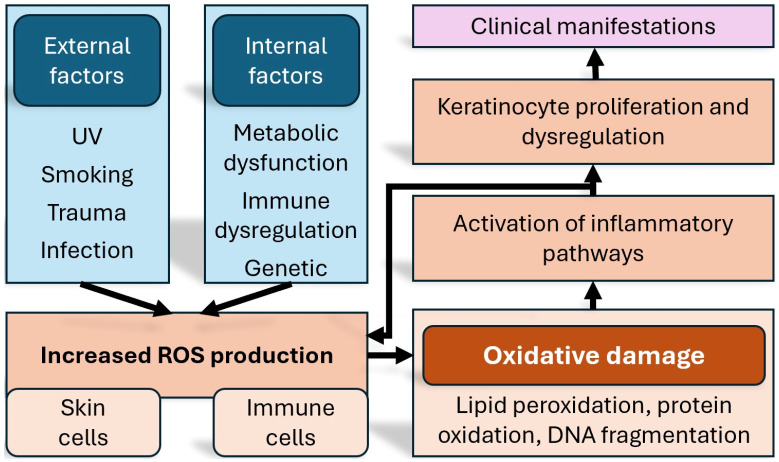
Schematic representation of the how oxidative stress affects inflammation, and the manifestations of psoriasis. ROS = reactive oxygen species. DNA = deoxyribonucleic acid.

While there is a genetic component in psoriasis, not all patients carrying the implicated genes will develop the disease. Perhaps the most prominent genetic associations at the moment are genes of the HLA complex [15]. It becomes therefore apparent that since genetic determinism alone cannot justify on its own the pathogenetic model, the onset of the disease must be related to environmental factors as well. It is also well established that the course and severity of the disease are influenced predominantly by environmental rather than intrinsic factors [16,17]. Another important contributing factor, based on recent research, is an increased amount of oxidative stress, which leads to the activation of a number of proinflammatory signalling pathways [18] (Figure 1).

One of the distinguishing features of psoriasis is the exaggerated production of reactive oxygen species (ROS) inside keratinocytes and immune cells [19]. The generated redox imbalance aggravates the inflammation via signalling pathways such as MAPK, STAT3, and NF-κB and contributes not only to local skin lesions but also to systemic comorbidities [20,21]. Cutaneous oxidative stress is generated in response to inflammatory cytokines such as IL-17 and TNF-α, leading to lipid peroxidation, protein oxidation, and DNA fragmentation; in contrast, systemic oxidative stress appears as an extension of the chronic inflammatory process beyond the skin, and the increased pro-inflammatory cytokine levels can negatively affect endothelial cells, leading to dysfunction [22,23].

Aside from medical interventions, the environmental factors implicated are psychological stress, substance and tobacco use and abuse, and diet. By themselves, these factors have been studied for decades by numerous researchers trying to establish causal relationships between diseases and risk factors. Indeed, physiological and psychological stress is now seen as being associated with both mortality and morbidity [24,25,26]. Smoking and excessive alcohol consumption have a host of research data demonstrating their importance as risk factors [27,28,29,30,31,32], while the role of diet in health maintenance and disease prevention has come into focus in the 21st century [33,34,35,36,37,38].

In the specific case of psoriasis, it seems that the principal associated comorbidity is obesity [39,40,41,42,43]. Obesity increases the risk of psoriasis incidence, while the cytokine profile in patients with metabolic syndrome and obesity and psoriatic patients is similar [44,45]. Obesity is more likely to develop in people following a sedentary lifestyle [46,47] and with high-fat diets [48,49]; dietary factors, both in the context of obesity and on their own, can modify disease progression and severity in the case of psoriasis.

In the last decades medical innovations have led to the development of novel therapeutic strategies [50]. Given the recent focus on the influence of nutrition and oxidative stress in psoriatic patients, the focus of this review will be the presentation of how diet, both from a quantitative and a qualitative perspective, influences the onset and severity of psoriasis, and via which mechanisms such influences are exerted.

## 2. Materials and Methods

In endeavoring to gather a complete and representative sample of research on psoriasis and nutrition, we have performed a thorough search in the most complete and widely used medical databases, namely PubMed, Scopus, Science Direct, and Medline. The search strategy relied on the terms “psoriasis”, “diet”, “obesity”, “oxidative”, “oxidants”, “antioxidants”, and “inflammation” in different combinations so as to cover as much probable relevant research material as possible, using the appropriate Boolean operators. For a paper to be considered as appropriate, the search terms should have to be included either in the title or abstract, preferably both. We tried different combinations of these keywords to ensure, as much as possible, the inclusion of all possible relevant papers, and we subsequently removed from our selection papers that did not exclusively deal with psoriasis but with oxidative stress in the context of autoimmune diseases. We have further excluded papers published in languages other than English, manuscripts not published in peer-reviewed journals, and those with a research question or patient population that were not relevant for our study.

Even though psoriasis was first described in modern medical terminology in the 18th century [51], the role of diet in its evolution and management began to be assessed after the 1950s, and research has only specifically focused on this aspect after 2010. Therefore, we have elected to confine our search to papers published between 2014 and 2024; the starting year has been selected specifically due to the relevant WHO resolution [52], which raised awareness of psoriasis and served as an impetus for intensifying research efforts. We have chosen to include, wherever possible, reviews that delve into mechanisms and associations, but where original papers reported essential or novel information, we have included them as well.

## 3. The Interplay Between Psoriasis and Nutrition

The profound influence of diet on human health and disease [53] has begun to be understood in the last decades [38,54]. At the same time, a notable majority of adults in the Western world suffer from at least one chronic disease, whose evolution and even risk of incidence may be associated with dietary factors [55,56].

At the same time, several different diet patterns have been implicated in the management of different diseases. For example, ketogenic diets may be useful in the management of neurological conditions [57]. Furthermore, nutritional supplementation may have potential in inflammatory biomarker control [58], and certain nutritional products may be useful in managing macular degeneration [59,60]. Other authors have focused on the role of gut microbiota in disease [61,62]. All these and other factors have been implicated in the incidence and management of psoriasis. As such, in this section, we will explore the role of oxidative stress, of diet and nutritional choices, of other supplements in general, and of vitamin D supplementation in particular.

### 3.1. The Role of Oxidative Stress in the Pathogenesis of Psoriasis

Oxidative stress can be defined as an imbalance manifesting as a temporary or chronic increase in the levels of free oxygen/nitrogen radicals resulting either from an increased production or a decrease in the ability of antioxidant systems to eliminate them [63], contributing to the pathogenesis of psoriasis.

Oxidative stress levels are measured by markers such as malondialdehyde (MDA), total oxidative stress (TOS), oxidative stress index (OSI), catalase (CAT), myeloperoxidase (MPO), ferroxidase (FOX), ischemia-modified albumin (IMA), paraoxonase-1 (PON-1), total antioxidant status (TAS), and 8-hydroxy 2′-deoxyguanosine (8H2D) [18,64]. Other useful markers include adipokines, namely adiponectin, leptin, visfatin, and resistin, due to their immunomodulating action [63,65], as well as urinary biopyrins [63,66]. There exist correlations between these markers and the severity of psoriasis, as measured by the Psoriasis Area and Severity Index (PASI) [18,63,66]; higher oxidative stress levels were associated with increased PASI scores. Out of all of them, the strongest correlation was found to exist between MDA levels and PASI, highlighting its potential as a biomarker for assessing psoriasis severity [67]. Thus, these findings support the theory that oxidative stress plays a crucial role in the development and complications of psoriasis.

Antioxidant enzymes, such as superoxide dismutase (SOD) and CAT, play a crucial role in reducing ROS levels, which are implicated in the pathogenesis of psoriasis [68,69,70]. A deficiency in these enzymes can lead to oxidative stress, contributing to inflammation and hyperproliferation of keratinocytes [43]. Therefore, maintaining or restoring the activity of these enzymes may offer therapeutic benefits in managing psoriasis symptoms [18].

In patients with active psoriasis, the levels of antioxidant enzyme levels, particularly SOD and CAT, are often reduced, indicating an impaired antioxidant defense system [18]. This deficiency can lead to increased oxidative stress, exacerbating inflammation and skin lesions associated with psoriasis. Consequently, monitoring and potentially restoring antioxidant enzyme levels may have therapeutic implications, offering a strategy to mitigate oxidative damage and improve disease management [18].

Another study emphasizes systemic inflammation through redox mechanisms, showing that oxidative stress associated with the development of psoriasis leads to oxidative protein changes, including a wide range of lipid peroxidation products [71]; the authors demonstrate that psoriasis-associated diseases can be effectively treated by inhibiting the formation of lipid peroxidation product–protein adducts and adjusting their concentrations during psoriasis therapy [71].

### 3.2. The Role of Diet in the Management of Psoriasis

Most studies agree that dietary patterns play a significant role in the management of psoriasis [40,70,72,73,74,75,76,77,78,79,80], as various skin diseases and conditions can be prevented or improved through changes in diet. Various dietary interventions, as well as single nutrients, can positively impact the clinical presentation, severity, and course of the disease [81]. Hypocaloric diets as well as antioxidant-rich diets can promote weight loss, reduce oxidative stress, and improve both the severity and the response of the condition to systemic treatments [40,73,76,82].

Since reducing caloric intake was found to alleviate symptoms [80,81], some forms of fasting, such as intermittent circadian fasting [74,83,84] and TRE (time-restricted eating) [78], may be promising in terms of their potential for symptom amelioration. This is in line with findings that suggest that intermittent fasting is associated with beneficial effects on autoimmune pathologies [74,83,84].

Generally, specialized diets, such as protein-restricted and vegetarian diets, may suppress systemic inflammation and inhibit angiogenesis [73], creating a less favorable environment for psoriasis. Diets rich in vegetables, with low or even zero animal protein intake, when combined with appropriate nutritional supplementation, seem to be associated with symptom amelioration in some patients, presumably due to their high fiber content and low saturated fat intake [73]. Although they may be wrongly characterized as risky for skin health due to nutritional deficiencies, such as lack of riboflavin (B2) and vitamin A, well-devised vegan diets can nevertheless meet nutritional needs and benefit inflammatory skin conditions like psoriasis, acne, and atopic dermatitis [85].

Elimination diets (e.g., gluten-free diet) may work for some patients, who report improvement by eliminating specific foods that may trigger flare-ups [82,86,87,88]. Common triggers include gluten-containing grains for those with gluten sensitivity or intolerance, dairy products, refined carbohydrates, nightshade vegetables (like tomatoes and potatoes), high-sugar foods, and processed foods [88]. However, these effects are individualistic; what triggers one person’s condition may not affect another. Consequently, when it comes to the totality of psoriatic patients, the benefits of a gluten-free diet are indeterminate [81].

The Mediterranean diet, which is rich in fruits and vegetables, provides polyphenols and antioxidants that help combat oxidative stress. Antioxidants such as flavonoids, vitamins A, C, and E, and β-carotene, along with oligo-elements like copper, manganese, zinc, and selenium, may offer protective effects against intrinsic skin damage [89]. Interestingly, it has been suggested that this diet’s beneficial effects can also be tied to changes in the gut microbiota [90]. Overall, greater adherence to the Mediterranean diet is linked to less severe forms of psoriasis and improved quality of life for patients [73,76,78,82,89,91,92]. The Mediterranean-like model tested by Castaldo et al. [93] on obese, drug-naïve patients in particular, consisting of a protein-sparing, very-low-calorie ketogenic diet, resulted in notable alterations of PASI scores. While the Mediterranean diet sensu stricto may be considered culturally specific to the Mediterranean region, its basic tenets and principles may be applicable in a wide range of populations, irrespective of their nationality or residential region. The effectiveness of the Mediterranean diet, but also of the ketogenic diet, in affecting psoriatic activity and the associated inflammatory markers is also corroborated by Lambadiari et al. [94] and Katsimbri et al. [95]. It is worth mentioning, however, that the ketogenic diet has been reported to have both ameliorating and exacerbating effects on psoriasis [96]. Apart from the presence of ambiguous data on this matter, it should also be mentioned that ketogenic diets have a number of potential adverse effects, including nutrient deficiency, fatigue, increased LDL cholesterol, or other chronic diseases [97,98]; therefore, a careful risk-benefit assessment should be undertaken on a patient-by-patient basis.

At the opposite end of the spectrum, there are the high-fat diets, like the Western diet, which may exacerbate the cutaneous manifestations, particularly through the action of saturated fatty acids (SFAs), such as palmitic acid, due to enhancement of skin inflammation via immune activation, independent of obesity [78,99].

Thus, this kind of fatty acid should be avoided in order to reduce psoriasis-related inflammatory lesions at the level of the skin [72]. The opposite is true of unsaturated fatty acids, namely monounsaturated (MUFA) and polyunsaturated (PUFA) fatty acids, which have been found to be able to decrease the risk of immunometabolic diseases. MUFAs, such as oleic acid, which are found in extra-virgin olive oil, safeguard lipoproteins and cell membranes from detrimental oxidative damage. PUFAs are divided into omega-3 and omega-6 fatty acids. Patients should be encouraged to increase their intake of omega-3 fatty acids (found in fish, flaxseed, and walnuts) since they can reduce inflammation [67,76,89]. Conversely, omega-6 fatty acids (commonly found in processed foods and vegetable oils) are promoters of inflammation, and consumption of the associated food sources should be restricted [43]. A cross-sectional study focusing on patterns of food consumption in psoriasis patients illustrates this point by assessing Pattern 1, consisting mostly of processed foods, and Pattern 2, being made up predominantly of fresh foods. Compliance with Pattern 2 was linked to normal serum lipids and blood pressure, a lower waist-to-hip ratio, and decreased psoriasis (skin) activity [100]. In the same vein, Clark et al. [101] conclude that the supplementation of omega-3 fatty acids leads to improvement of PASI score, as well as of the erythema and scaling, though it remains uncertain whether they can help with other signs and symptoms, like desquamation and itching. The beneficial nature of omega-3 fatty acids is also supported by the fact that the Mediterranean diet, the effectiveness of which was described previously, has a favorable omega-3 to omega-6 ratio [40].

As far as specific dietary instructions are concerned, according to the United States National Institutes of Health Panel [102], the ideal ratio of omega-3 to omega-6 fatty acids should be approximately 1:1.80, though other reports place it at a range of 1:30 to 1:50 [103,104,105].

Natural antioxidants can be used to treat inflammatory diseases, including psoriasis. Because of its anti-inflammatory and antioxidant properties, it has been discovered that the natural phytocannabinoid oil has been suggested as a treatment for psoriasis [106].

Similarly, it has been discovered that cannabidiol (CBD) can provide considerable protection from UV-induced oxidative stress to skin cells [107,108], as, after applying CBD to psoriatic skin, there was a decrease in proinflammatory mediators and relevant proteins [107], and it induces metabolic alterations in keratinocytes [109]; other authors report a decrease in keratinocyte proliferation [103,108]. Furthermore, psoriatic arthritis patients report less pain when using CBD [75,108]. Although there is insufficient evidence to confirm this, other antioxidant and anti-inflammatory substances, such as the lipid extract of microalgae [69], are proposed as possible anti-psoriatic factors, their actions being based on preventing lipid peroxidation products from interacting with proteins. Regarding CBD, it must be stressed that this is not a risk-free substance, and a host of adverse effects have been reported [110]; at the same time, more clinical trials on its use in psoriasis patients must be designed, with the aim of elucidating the full spectrum of its therapeutical value and its benefit-to-adverse-effect ratio. We must note that the aforementioned CBD applications are mostly researched at a cellular level in vitro; however, based on these encouraging results, several authors have explored its possible use as a supplement [111,112].

Anti-inflammatory effects can be exerted by adhering to diets that include foods such as fruits, vegetables, whole grains, fatty fish (rich in omega-3 fatty acids), nuts, and seeds [85,87,89]. This is made possible due to their ability to assist in lowering oxidative stress and modulating immune responses, having been associated with lower levels of inflammation markers, such as C-reactive protein (CRP) [87].

As mentioned before, obesity is a known risk factor for psoriasis. This is because adipose tissue produces adipokines, the proinflammatory action of which is implicated in the pathophysiology of the condition, being able to bring about exacerbations [79]; hence, this is why they are useful as markers [63,65]. As such, it can be said that adipokines play a crucial role in the relationship between obesity, psoriasis, and nonalcoholic fatty liver disease (NAFLD); adiponectin, a hormone that promotes insulin sensitivity and fatty acid oxidation, is closely associated with both psoriasis and NAFLD [113]. Therefore, weight loss or weight management through any of the aforementioned balanced diet patterns can lead to a decrease in blood serum inflammatory factors and consequently to significant improvements in psoriatic symptoms [40,42,70,75,78,87]. Since high alcohol consumption and smoking are associated with increased severity of psoriasis [105,114], studies also show that reducing or excluding these substances can lead to improvements in skin health and overall well-being [80,87].

### 3.3. The Role of Vitamin D in the Management of Psoriasis

Vitamins play a significant role in the treatment of mild to moderate psoriasis. Currently, various vitamins and their analogues are employed to manage these forms of psoriasis, either administered individually or in combination with other medications [68]. Vitamins are essential in psoriasis treatment, with two primary therapeutic vitamins and their derivatives being vitamin A and vitamin D [68].

Vitamin D, also called the sunshine vitamin, is mainly produced by the skin upon exposure to UVB radiation [115]. It is then hydroxylated in the liver to form 25-hydroxyvitamin D, or 25(OH)D3, an inactive form of the vitamin, and undergoes further hydroxylation in the kidneys to produce the biologically active form of vitamin D, calcitriol, or 1,25(OH)D3. In order to ensure the required amount of vitamin D, it is necessary to expose the forearm for 15–20 min to UV sunrays. A major problem is advanced technology, which induces people to spend more and more time indoors, and this leads to a significant decrease in vitamin D levels [116,117].

There are two main forms of this vitamin, ergocalciferol (vitamin D2) and cholecalciferol (vitamin D3); only vitamin D3 is the bioactive form [118]. Few foods naturally contain vitamin D, and they are primarily of animal origin [118]. Vitamin D2 is produced by plants, but fruits and vegetables in the human diet contain only minimal amounts. The best sources of vitamin D2 are nuts, whole cereals, and vegetables. Mushrooms provide variable amounts of vitamin D2, which can be enhanced through ultraviolet light exposure under controlled conditions [119]. In contrast, vitamin D3 is naturally found in certain animal foods, particularly meat, egg yolk, butter, pork liver, and fatty fish such as salmon, herring, and mackerel, as well as in fish oils [118].

Additionally, vitamin D3 is available through dietary supplements and is the form present in vitamin D-fortified foods such as milk, orange juice, and cereals [118]. Fortification with vitamin D is considered a promising and impactful strategy [120]. The recommended daily dose of vitamin D3 for adults between 19 and 50 is 600 UI/day, while for adults over 60, the International Osteoporosis Foundation recommends 800–1000 UI per day [41].

Vitamin D’s properties have been investigated for decades in the treatment of psoriasis [121], as it plays a crucial part in the maintenance of the homeostasis of the cutaneous barrier [118]. Various studies have explored the correlation between vitamin D-specific receptors and susceptibility to psoriasis [122,123]; it has been discovered that the A-1012G promoter polymorphism of the vitamin D receptor (VDR) gene is linked to an increased risk of psoriasis due to reduced expression of VDR mRNA, which may contribute to changes in the cutaneous barrier and the formation of psoriatic lesions [124]. Moreover, a reduced expression of VDR and decreased tight junction proteins are commonly observed on the skin of psoriatic patients [125,126]. Tight junctions are essential for regulating the adhesion and permeability of keratinocytes, polarizing cutaneous cell differentiation, and managing the extracellular calcium gradient. They interact with nuclear and cytoplasmic proteins and influence the regulation of specific genes involved in keratinocyte differentiation and proliferation [41]. Thus, maintaining the skin barrier homeostasis is essential for maintaining the tight junctions of epithelial and endothelial cells, which are under threat of being dysregulated due to psoriasis, and it reduces the pathological inflammatory response. As far as associated comorbidities are concerned, the study of Barrea et al. [41] suggests that vitamin D deficiency in psoriasis may be linked to both obesity and cardiovascular disease.

Topical vitamin D is commonly prescribed, either alone or with topical corticosteroids, for localized plaque psoriasis, showing good results [127]. Especially when combined with vitamin D analogues, corticosteroids are more effective and provide remission for longer periods of time when compared to monotherapy [94]. Those analogs are especially useful for sensitive areas like the face and do not lead to tolerance like corticosteroids do. They can be used indefinitely without serious side effects and are effective for both children and the elderly [41]. A recent analysis showed that topical vitamin D treatments have similar effectiveness to corticosteroids and even better results when combined with potent steroids, showing a “steroid-sparing” effect [118]. That being said, the findings of Ford et al. [81] suggest that oral vitamin D supplementation can help with psoriatic arthritis but not with the skin-related manifestations. In a similar vein, according to Formisano et al. [128], vitamin D supplementation does not affect PASI values in any notable way. Despite not affecting the PASI values, vitamin D deficiency is considered a risk factor for psoriasis [128], and, as mentioned earlier, while the cutaneous manifestations of psoriasis may be more concerning from the point of view of quality of life, other manifestations of the disease may benefit from such an intervention.

At any rate, in addition to its topical application, oral vitamin D supplementation serves as an important adjunctive treatment option for patients with psoriasis [118]. Secondly, vitamin D supplementation may play a crucial role in the prevention of psoriasis-related comorbidities, including hypertension [129] and metabolic syndrome [120], the latter being correlated with vitamin D deficiency [130]. Furthermore, its derivatives are capable of ameliorating the efficacy of phototherapy without causing any unwanted side effects [131].

### 3.4. The Role of Dietary Supplements in Psoriasis

Due to the potential side effects and benefits of simultaneous conventional and complementary and alternative medicine (CAM) practices, alternative or integrative therapies that may serve as substitutes or supplements to conventional treatment should be explored. The positive impact of macro- and micronutrient supplementation on improving psoriasis should be taken into account. The most frequently utilized oral dietary supplements among psoriasis patients are fish oil, selenium, and zinc [132]. Studies show that the evidence for zinc supplementation is controversial, yet supplements such as fish oil (omega-3 fatty acids) and selenium have been found beneficial for psoriasis patients. Results on the effectiveness of fish oil supplementation in psoriasis are ambivalent, with some reviews demonstrating a benefit [67] and some a lack of effectiveness [133]. The dosages varied, with the average being 4 g/day of eicosapentaenoic acid (EPA) and 2.6 g/day of docosahexaenoic acid (DHA). These supplements should be taken for an extended period (1–6 months) to achieve a noteworthy improvement in psoriasis. It was shown that consuming 6 ounces (170 g) of fatty fish daily improved psoriasis compared to the intake of white fish [67].

As for vitamin D, studies reveal that while topical vitamin D is effective [79,89] in counteracting inflammation at the skin level [79], oral supplementation is not recommended for psoriasis treatment in adults with normal vitamin D levels [73]. As mentioned before, vitamin D supplementation may be beneficial in selected cases, particularly in those with documented hypovitaminosis to prevent psoriasis-related comorbidities [76]. Topical application of phytoestrogens like genistein, present in soybeans, ameliorated the symptoms of psoriasiform dermatitis [99].

In addition, certain phytochemical compounds like *Dunaliella bardawil* (the richest known source of the antioxidant β-carotene), *Tripterygium wilfordii* (which contains bioactive triptolides and terpenoids), *Azadirachta indica* (the neem tree), and *Curcuma longa* (turmeric) are worth mentioning. Similarly, HESA-A is a compound produced in accordance with traditional Persian medicine, comprising mineral, herbal, and animal (marine shrimp) components, which has exhibited important benefits in clinical trials for improving psoriasis severity [73]. It is predominantly an herbal formulation, with all of its constituents being derived from the Apiaceae family; *Kelussia odoratissima* Mozaff. (wild celery) is paired with *Cuminum cyminum* L. (cumin), while *Apium graveolens* L. (cultivated celery) is paired with *Carum carvi* (*C. carvi*) L. (wild cumin) [73]. While more research is required in such traditional medical schemes, from the point of view of documenting their efficacy and their dosage and toxicity profiles, specifically in the case of HESA-A, there is a host of data documenting its use in certain pathological states [134,135].

Finally, prebiotics and probiotics may help by reducing lipopolysaccharide production and promoting a healthy gut microbiota, which can decrease inflammation [76,89,99,136,137,138].

## 4. Discussion

From all the aforementioned, it is evident that there exist numerous extrinsic factors that can be modified in the context of psoriasis management. Perhaps the most important aspect, from the point of view of pathophysiology and inflammation, is oxidative stress. Oxidative stress, which can be caused by numerous factors [139,140,141,142], is under research for its importance in several different pathologies [143]. Based on current evidence, in psoriatic patients there is an impairment of most antioxidant mechanisms [18], and the adjustment of lipid peroxidation may lead to improved outcomes [71]. Regarding the role of diet, there are several interventions that can contribute to more favorable outcomes, such as phytocannabinoid oil intake [106], reduced calorie intake, or elimination diets; specialized diets may be of benefit for some patients (Table 1).

Probably the most important role may be that of substances, phytochemicals in particular, which have concurrent antioxidant and anti-inflammatory properties; some such compounds have already been explored as a potential management solution [73]. Other such phytochemicals may be tried in the management of psoriasis, like kaempferol [144,145], pinosylvin [146], compounds derived from black pepper [147,148], capsaicin [149,150], and thymol [151]. Other recent concepts concern the role of probiotics and of gut microbiota in inflammation and disease [75,137,152,153]; the modification of gut microbiota may be useful in the management of psoriasis [136,154,155,156,157] and surely represents an interesting avenue for future research. Intestinal microbial dysbiosis has already been documented in psoriatic patients [158,159], while the dysregulation of the skin microbiome has also been proposed as a contributing factor [160].

In the introduction we have mentioned the influence of certain risk factors such as smoking and stress on mortality and morbidity. Stress and obesity are interconnected in many cases [161,162], and smoking often coexists with higher body mass index (BMI) [163,164], even though this relationship is not causal. Therefore, it can be seen that there is, most likely, an interplay between all these factors, directly or indirectly [165]; stress leads to psoriasis exacerbation [166], in turn leading to more stress [167], in a vicious cycle scheme. Therefore, the need for multidisciplinary management [168], including psychological management, in such patients becomes apparent. Similar multidisciplinary approaches have already been successfully tried in psoriatic arthritis [169,170]. Apart from such factors, the multidisciplinary aspect should be focused on other aspects such as joint involvement in psoriasis [171]; advanced modern concepts such as 3D printing applications [172,173,174,175] can be considered in such cases.

A proper diet and exercise regimen will lead, in the absence of other metabolic pathologies, to weight loss and the maintenance of a healthy physique. This is important when considering the interplay between obesity and inflammation [176,177]. Even in psoriatic patients, where there is persistent systemic inflammation, it follows that weight loss will be correlated with an improvement of disease status. Finally, the role of hormonal imbalances and treatment in psoriasis must be considered. In particular, hormonal imbalances may alter the presentation and clinical course of psoriasis [178,179], while hormonal treatment may be effective. For example, somatostatin treatment has been tried in the past with mixed results [180,181]; perhaps the use of more modern somatostatin analogs [182,183] should be more eagerly investigated in the future.

Even though the pathophysiology of psoriasis is extremely complex [184,185], there exists a host of modifiable factors that can influence its development, severity, and impact on quality of life. Altering dietary habits can greatly enhance the quality of life for patients, benefiting both psoriatic lesions and decreasing the likelihood of other associated diseases. Though there are no national or international guidelines recommending a specific nutritional approach to the management of psoriasis, several approaches may be tried based on the data reported in this review, either as a general rule or on an individualized patient-by-patient basis.

In considering the clinical significance of all the data herein presented, we must, at first, emphasize that while the data on the reduction of oxidative stress using dietary interventions are promising, in most cases there are additional steps which that be taken in order to obtain statistically significant correlations in wide and diverse patient samples. On the other hand, the wealth of encouraging data enables us to suggest that, pending further research, the implementation of dietary interventions in a patient-by-patient case may be of use at the moment. However, in certain cases, for example, when implementing ketogenic diet schemes or administering herbal or traditional supplements, the medical personnel should be mindful of potential adverse effects or toxicity.

The improvement in patient quality of life may be further increased by stress management and the elimination or reduction of other risk factors, which have already been presented. Modifying such factors, especially based on patient decision, may lead to self-empowerment, which can lead to better treatment outcomes [186,187].

## 5. Conclusions

It can be concluded that the management and clinical course of psoriasis, while in part depending on genetic factors, is mostly attributable to extrinsic factors. An important aspect of such factors is nutrition; in the context of nutrition, there are various approaches that have been tried, ranging from adopting different diet patterns to altering caloric intake or administering supplements. Perhaps the most crucial implicated factors, which can be modified by said interventions, are oxidative stress and obesity. Both are linked to and influence inflammation, which is arguably the most prominent component in the majority of psoriasis cases. Future studies should aim to expand on quantifying the influence of such factors in the management and quality of life of such patients and on examining the potential beneficial effects of other antioxidants, namely phytochemicals, in oral or topical administration.

## Figures and Tables

**Table 1:** Dietary and nutritional interventions and supplements, and their effects on psoriatic patients, and some foods or dietary patterns that aggravating the disease are also presented.

Type of Intervention/Diet Scheme	Results	Year	Reference
**General Diet and Weight Loss Interventions**			
Weight loss via caloric restriction or surgery	Improvement of disease status in many patients in conjunction with standard medical treatment	2014	[42]
Weight loss via caloric restriction and increase in unsaturated fats and antioxidant intake	Improvement of disease status and general health condition of patients	2015	[114]
Weight loss via different diet patterns	Increase in drug pharmacokinetics and efficiency and general improvement of patient condition	2016	[40]
Weight loss via different diet patterns	Increase in treatment efficacy and improvement in the cardiovascular profile of the patients	2016	[113]
Weight loss via diet modifications and a healthy lifestyle	Improvement in PASI scores	2018	[73]
Weight loss via caloric restriction in overweight or obese patients	Probability of reduction in disease severity	2018	[81]
Gluten-free diet only in patients with a proven gluten sensitivity	Probably improvement in patient state		
Intermittent circadian fasting	Positive impact on psoriasis and psoriatic arthritis	2019	[74]
Adherence to a Mediterranean diet scheme	Decrease in a subjective and objective severity of psoriasis	2019	[91]
Predominance of fresh instead of processed foods in diet	Lower skin disease activity in many patients	2020	[100]
Weight loss and adoption of the Mediterranean diet patterns	Decrease of inflammation and improvement in patient state	2021	[95]
Adoption of the Mediterranean diet	Improvement in patient status in conjunction with standard medical treatment	2022	[76]
Adoption of the Mediterranean diet	Decreased severity of psoriasis and psoriatic arthritis	2023	[82]
Intermittent fasting patterns	Possible effects in psoriasis and other autoimmune disorders of inflammatory nature	2023	[84]
Adoption of the Mediterranean diet	Potential anti-inflammatory benefit	2024	[92]
Combination of ketogenic and Mediterranean diets	Reduction of inflammatory and disease markers	2024	[94]
**Nutritional Supplementation**			
Supplementation with Zn, Se, Ω3, HESA-A, and other botanical species	Various benefits in psoriatic patients (depending on the supplements, patient condition, etc.)	2018	[73]
Intake of proanthocyanidins (along with possible topical application)	Possible correction of Th17/Treg cell imbalances, keratinocyte overproliferation, and angiogenesis	2018	[87]
Ω3 supplementation	Improvement of disease parameters in psoriatic patients	2019	[101]
Supplementation with oral probiotics	Potential benefits in conjunction with standard medical therapy	2022	[76]
**Vitamin D Supplementation**			
Oral vitamin D supplementation in deficient patients	Prevention of psoriasis-related comorbidities	2018	[73]
Oral vitamin D supplementation in conjunction with weight loss	Possible benefits to patients status if used along with standard medical treatment	2018	[81]
**Local In Vitro Cannabidiol Applications**			
Cannabidiol application in irradiated keratinocytes	Reduction of oxidative stress in cells isolated from healthy individuals—possible protective effect	2020	[109]
Cannabidiol application in 3D-cultured keratinocytes	Protective effect of cannabidiol against irradiation	2021	[107]
Cannabidiol application in irradiated keratinocytes	Possible protective effect against irradiation	2021	[108]
**Aggravation of Psoriasis**			
Increased alcohol consumption	Increased severity of psoriasis manifestations	2017	[105]
Increased saturated fatty acid intake	Increase of skin inflammation	2018	[72]
Increased BMI	Increased chances of psoriasis incidence	2019	[83]
Western-style diet, rich in sugars and fats	Promotion of a pro-inflammatory state possibly due to gut microbiota dysregulation	2021	[75]
Western-style diet, rich in sugars and fats	Immune aberration in the production of pro-inflammatory cytokines	2021	[95]
Poor dietary habits and alcohol abuse	Increased chance of psoriasis incidence or increase in disease severity	2023	[80]

## Data Availability

Not applicable.

## Abbreviations

The following abbreviations are used in this manuscript:

25(OH)D3	25-hydroxyvitamin D
8H2D	8-hydroxy 2′-deoxyguanosine
CAM	Complementary and alternative medicine
CAT	Catalase
CBD	Cannabidiol
CRP	C-reactive protein
DHA	Docosahexaenoic acid
EPA	Eicosapentaenoic acid
FOX	Ferroxidase
HLA	Human leucocyte antigen
IMA	Ischemia-modified albumin
MDA	Malondialdehyde
MPO	Myeloperoxidase
MUFA	Monounsaturated fatty acids
NAFLD	Nonalcoholic fatty liver disease
OSI	Oxidative stress index
PASI	Psoriasis area and severity index
PON-1	Paraoxonase-1
PUFA	Polyunsaturated fatty acids
SFAs	Saturated fatty acids
SOD	Superoxide dismutase
TAS	Total antioxidant status
TOS	Total oxidative stress
VDR	Vitamin D receptor
WHO	World Health Organization
